# Supplementary material for: Exploring Photoreceptor Gene Expression and Seasonal Physiology in Mediterranean Swordfish (Xiphias gladius)
Source: Animals (Basel). 2024 Nov 14;14(22):3273. doi: 10.3390/ani14223273 (PMC11590907; doi:10.3390/ani14223273)
Supplement: Supplementary file 1 [file animals-14-03273-s001.zip › animals-3164359-supplementary.pdf]

Table S1. Relative gene expression. Data reported as mean values ± SD.

Gene symbol	Immature females	Mature females
asmt	10.650 ± 8.583	19.120 ± 14.990
mel1b	8.284 ± 14.940	66.870 ± 71.760
sws	186.6 ± 135.6 [a,1]	20.62 ± 29.70 [b]
opsin4	1.278 ± 1.946	5.049 ± 9.704
VA opsin	19.37 ± 18.17	6.831 ± 3.071
sod1	4.648 ± 3.730	7.113 ± 3.280
sod2	5.478 ± 4.303	11.890 ± 7.291
hspa4b	3.703 ± 2.242	10.010 ± 5.227

[1]Different letters indicate a significant difference.

Table S2. Histological analysis. Data reported as mean values ± SD.

	Immature females	Mature females
Area of MMCs (μm²/mm²)	4164 ± 2570 [a,1]	17295 ± 6951[b]
Number of MMCs (#/mm²)	21.77 ± 12.63 [a]	36.93 ± 14.17 [b]
Number of MMs (#/mm²)	59.36 ± 22.83 [a]	102.0 ± 31.24 [b]
Lipid portion μm²/mm²)	969528 ± 373384 [a]	670231 ± 187256 [b]

[1]Different letters indicate a significant difference.
